# Hypopituitarism due to a Large Osteoclastoma Arising from the Sphenoid Bone Invading the Pituitary Fossa in a Patient with Parathyroid Carcinoma

**DOI:** 10.1155/2023/8274108

**Published:** 2023-12-21

**Authors:** Leonardo Bandeira, Lucian Batista de Oliveira, Maria Vitória Silva de Lima, Daniella Rêgo, Luiz Griz, Francisco Bandeira

**Affiliations:** ^1^FBandeira Endocrine Institute, Recife, Brazil; ^2^Grupo Fleury, Recife, Brazil; ^3^Division of Endocrinology, Diabetes and Metabolic Bone Diseases, Agamenon Magalhães Hospital, University of Pernambuco Medical School, Recife, Brazil

## Abstract

**Background:**

Parathyroid carcinoma accounts for <1% of cases of primary hyperparathyroidism (PHPT). This rare condition may present with severe hypercalcemia and bone complications such as osteoclastomas and pathologic fractures. Here, we present a rare condition of panhypopituitarism resulting from an osteoclastoma in the sphenoid bone that invaded the pituitary fossa due to parathyroid carcinoma. *Case Report*. A 47-year-old woman previously diagnosed with PHPT underwent a parathyroidectomy 6 years earlier, with histological examination indicating a parathyroid adenoma. After surgery, she continued to exhibit high serum parathyroid hormone (PTH) and calcium levels, with the development of bone pain and spontaneous fractures. Imaging exams showed a large osteoclastoma of the sphenoid bone, invading the pituitary fossa, causing hypopituitarism. A new parathyroidectomy was performed, with histological confirmation of parathyroid carcinoma and regression of the osteoclastoma.

**Conclusion:**

This case illustrates an unusual presentation of parathyroid carcinoma, in which an osteoclastoma of the sphenoid bone caused hypopituitarism.

## 1. Introduction

Primary hyperparathyroidism (PHPT) is characterized by elevated parathyroid hormone (PTH) levels associated with elevated serum calcium. Single benign parathyroid adenoma accounts for 85% of cases, followed by multiple adenomas and hyperplasia. Parathyroid carcinoma accounts for less than 1% of cases of PHPT [1–3].

Parathyroid carcinomas are large and infiltrative, commonly causing invasion of surrounding structures [4, 5]. Its diagnosis is challenging, usually being histologically indicated by the presence of invasion of adjacent tissues or distant metastases [4–6]. Most cases of parathyroid carcinoma cause very high PTH levels and severe hypercalcemia, with renal and bone complications, such as *osteitis fibrosa cystica*, osteoclastomas, and pathological fractures [4, 6]. Rare cases may have a slow progression and manifest metastatic disease years after the surgical procedure [5–7].

This study reports a case of severe PHPT due to parathyroid carcinoma, with an osteoclastoma atypically localized in the sphenoid bone, affecting the sella turcica (pituitary fossa) and causing hypopituitarism.

## 2. Case Presentation

A 47-year-old woman was admitted to our endocrine clinic presenting severe bone pain and a history of bilateral spontaneous hip fractures. She had been diagnosed with PHPT six years earlier and underwent a right upper parathyroidectomy, with histopathological examination indicating parathyroid adenoma.

After surgery, she continued to exhibit high serum PTH and calcium levels, in addition to spontaneous fractures that generated deformities in the left lower limb and hip replacement surgery on the right. She has also reported a weight loss of about 10 kg over the past year, associated with weakness and hair loss, as well as impaired peripheral vision. The ophthalmological examination revealed bitemporal hemianopsia, with maintenance of visual acuity and no changes in the optic fundus.

Upon admission to our hospital, laboratory tests showed serum PTH of 3200 pg/mL, total calcium of 14.5 mg/dL, albumin of 2.4 g/dL, alkaline phosphatase of 1224 U/L, lactate dehydrogenase (LDH) of 598 U/L, blood urea nitrogen (BUN) of 21 mg/dL, serum creatinine of 0.8 mg/dL, hemoglobin of 8.4 g/dL, hematocrit of 25.7%, serum phosphate of 2.4 mg/dL, and 25-hydroxyvitamin D (25OHD) of 6.9 ng/mL.

Neck ultrasound showed a 2.2 cm nodule located adjacent to the inferior part of the right thyroid lobe and another one located adjacent to the superior part of the same lobe, measuring 1.9 cm, showing a marked uptake in technetium-99m sestamibi scintigraphy. There was also a large kidney stone located at the right renal pelvis. Distal 1/3 radius bone mineral density was 0.357 g/cm^2^ and the T-score was −6.0 SD.

**Figure 1:**
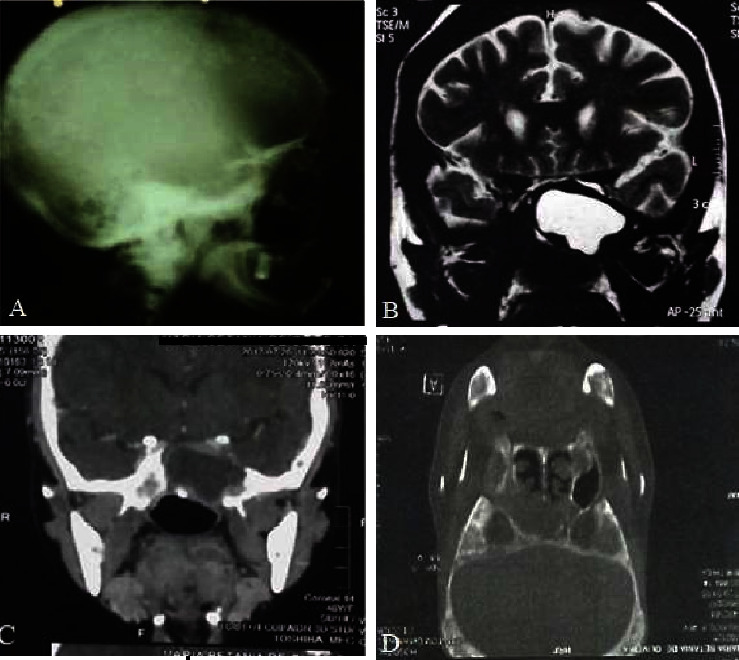
(A) Skull X-ray: enlargement of the sella turcica. (B) Skull MRI in T2 and (C) skull MRI in T1: presence of a 3.1 cm lesion invading the pituitary fossa. (D) MRI in T1: infiltrative lesion compromising the bony segments of the skull and face.

**Figure 2:**
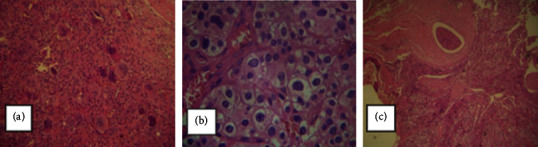
Histology of osteoclastoma in sphenoid sinus and parathyroid carcinoma. (a) Proliferation of multinucleated giant cells (osteoclast-like). (b, c) Areas of necrosis, capsule invasion, peripheral adipose tissue and cell pleomorphism. Immunohistochemistry: positive antibodies MRQ-31, Ki-67, galectin-3, and CD31.

Skull X-ray showed a *salt and pepper* appearance, *lamina dura* resorption on the central lower teeth, and enlargement and impairment of the pituitary fossa. The magnetic resonance imaging (MRI) showed a 3.1 cm contrast-enhanced mass invading the pituitary fossa and causing compression of the optic chiasm (Figure 1). In view of these findings, the pituitary function was evaluated, observing the following results: adrenocorticotropic hormone (ACTH) <5 pg/mL, free thyroxine (FT4) 0.67 ng/dL, thyroid-stimulating hormone (TSH) 0.52 mU/L, luteinizing hormone (LH) 14.98 mUI/mL, stimulating hormone follicle (FSH) 2.0 mUI/mL, prolactin (PRL) 7.45 ng/mL, basal cortisol 6.6 *μ*g/dL, and insulin tolerance test (ITT) with a peak cortisol level of 7.9 *μ*g/dL. A transsphenoidal biopsy histologically confirmed an osteoclastoma located at the sphenoid bone (Figure 2(a)).

In a new surgical approach, nodules located adjacent to the thyroid were excised, with the histopathological examination demonstrating neoplasia consisting of diffuse proliferation and rarely cell nests, mitotic figures, necrosis, capsular, and vascular invasion with emboli (Figures 2(b) and 2(c)). Immunohistochemistry showed positive antibodies to MRQ-31, Ki-67, galectin-3, and CD31. In the perioperative period, the patient received intravenous hydrocortisone therapy, with weaning during hospitalization. She also received levothyroxine 50 mcg/day. In the preoperative period, intravenous hydration and pamidronate were administered, aiming to reduce serum calcium. She was kept under intensive monitoring and did not present hypocalcemia due to bone hunger syndrome.

**Figure 3:**
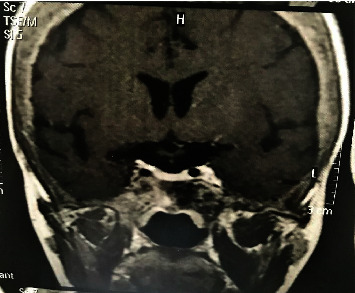
MRI in T1 showing significant regression of pituitary fossa lesion one year after parathyroidectomy.

After one year, the patient showed marked clinical improvement, especially in well-being, although PTH decreased but remained elevated, with serum calcium reaching levels at the upper limit of normal, characterizing a normocalcemic hyperparathyroidism. Pituitary MRI showed shrinkage of the sphenoid bone osteoclastoma (Figure 3), and new laboratory tests showed normalization of the pituitary corticotroph function (Table 1).

## 3. Discussion

We report a rare case of parathyroid carcinoma, with severe hypercalcemia and involvement of target organs, in which the emergence of an extensive brown tumor in the sphenoid bone, which invaded the pituitary fossa and caused hypopituitarism, was highlighted.

Parathyroid carcinoma is a rare cause of PTH-related hypercalcemia, with clinical features consistent with the symptomatic PHPT phenotype, usually with significant hypercalcemia and renal and bone involvement [3, 6]. Symptoms such as bone and muscle pain, fractures, urolithiasis, and hypercalcemic crisis are frequent [6]. More commonly, it is an isolated disorder, but it can be associated with hereditary syndromic conditions such as multiple endocrine neoplasia (MEN) 1, MEN 2A, isolated familial hyperparathyroidism, and hyperparathyroidism-jaw tumor [4, 6]. Its diagnosis is difficult, usually being confirmed in the histopathological examination, when infiltrative growth is observed, with the presence of vascular, lymphatic, and/or perineural invasion, invasion of adjacent anatomical structures, and/or metastatic disease [5, 6, 8]. Some immunohistochemical markers, such as the parafibromin, Ki-67 proliferation index, and galectin-3 can help in the diagnosis [6, 8]. In the present case, the histopathological findings of the first operation, in another service, were evaluated as a single parathyroid adenoma, but the patient presented a continuation of an exuberant picture of hyperparathyroidism, being repaired six years later, when she arrived at our service, when findings compatible with parathyroid carcinoma on histopathology (such as capsular invasion) and immunohistochemistry (Ki-67 and galectin-3).

Osteitis fibrosa cystica is a rare disease and occurs in less than 5% of PHTP due to excessive osteoclast activity. It characteristically presents with severe bone involvement such as salt and pepper lesions in the skull, subperiosteal bone resorption, distal clavicle tapering, bone cysts, and brown tumors, also known as osteoclastomas [9, 10]. Brown tumors are described as cystic lesions with a lithic aspect, benign, uni- or multifocal, filled with fibrotic tissue and granulation, typically with giant cells and other fusiform cells, containing hemorrhagic foci that allow the formation of hemosiderin deposits, leading to a brown coloration [10–12]. Brown tumors may manifest causing edema, bone pain, and spinal fracture. Due to the presence of spindle cells and giant cells mixed with poorly mineralized bone tissue and fibrous tissue, histology is not always specific because other bone tumors such as giant cell tumors and aneurysmal bone cysts have a similar histologic pattern. In these cases, to confirm whether the lesion is secondary to hyperparathyroidism, biochemical examination is required, in which the increase in serum calcium level may occur in association with the increase in PTH. With parathyroidectomy and improvement of hyperparathyroidism, bone lesions tend to regress [9–12]. They can be found in any bone sites of the skeleton, being most commonly found in ribs, clavicles, spine, skull, and hip long bones [9, 11, 12]. However, atypical locations such as the sellar and parasellar region and sphenoid bone have been reported previously in one case [13], but without pituitary involvement. This is another aspect that makes the present case unusual, since, to the best of our knowledge, there has been no previous report of hypopituitarism due to invasion of the adenohypophysis by an osteoclastoma. The patient had a 3.1 cm lesion in the sphenoid bone, histologically confirmed as a brown tumor, which caused invasion of the sellar and suprasellar region, with bitemporal hemianopsia and impairment of pituitary function, but without other compressive symptoms, such as headache, amaurosis and proptosis. After parathyroidectomy, the patient presented regression of the osteoclastoma and recovery of pituitary corticotroph function, with normalization of serum calcium and persistence of high PTH levels (which declined from approximately 50 times the normal range to 30 times). These findings indicate a persistent parathyroid carcinoma, although with partial remission, which provided substantial clinical improvement. The relevant decline in serum calcium sustained one year after surgery, reaching the upper limit of normal when corrected by albumin, helps to explain the involution of osteoclastoma that occurred after reducing tumor burden with the parathyroidectomy, as resistant hypercalcemia is an important adverse clinical prognostic factor in parathyroid carcinoma [6].

## 4. Conclusion

In conclusion, this case illustrates an unusual presentation of severe PHPT due to parathyroid carcinoma, in which an osteoclastoma of the sphenoid bone caused hypopituitarism.

## Figures and Tables

**Table 1:** Laboratory tests before and one year after surgery.

Laboratory test	Result before parathyroidectomy	Result one year after parathyroidectomy	Reference values
*Biological parameters*			

Hemoglobin (g/dL)	8.4	11.1	12 to 15.8

Hematocrit (%)	25.7	33.8	33 to 47.8

Creatinine (mg/dL)	0.8	0.5	0.8 to 1.5

BUN (mg/dL)	21	19	17 to 49

LDH (U/L)	598	—	135 to 214

Total calcium (mg/dL)	14.5	9.5	8,4 to 10.2

Alkaline phosphatase (U/L)	1224	780	35 to 104

Albumin (g/dL)	2.4	3.1	3.5 to 5.2

Phosphate (mg/dL)	2.4	2.7	2.5 to 4.5

*Hormonal parameters*			

PTH (pg/mL)	3200	2068.7	19 to 65

25OHD (ng/mL)	6.9	11	Deficiency:<20
			Insufficiency: 21 to 29
			Sufficiency: ≥30

Basal cortisol (*μ*g/dL)	6.6	19.7	6.7 to 22.6

ACTH (pg/mL)	<5.0	—	up until 46

LH (mU/mL)	14.98	46	Follicular fase:2.12 to 10.89
			Ovulatory phase: 19.18 to 103.3
			Luteinic phase: 10.87 to 58.64
			Postmenopausal woman 10.87 to 58.64

PRL (ng/mL)	7.45	7.40	2.8 to 29.2

FSH (mU/mL)	2.0	115	Follicular fase:3.85 to 8.78
			Ovulatory phase: 4.54 to 22.51
			Luteinic phase: 1.79 to 5.12
			Postmenopausal woman: 16.74 to 113.59

FT4 (ng/dL)	0.67	0.58	0.7 to 1.8

TSH (mU/L)	0.52	3.83	0.45 to 4.12

25OHD: 25-hydroxyvitamin D, ACTH: adrenocorticotropic hormone, BUN: blood urea nitrogen, FT4: free thyroxine, LDH: lactate dehydrogenase, LH; luteinizing hormone, PRL: prolactin, PTH: parathyroid hormone, and TSH: thyroid-stimulating hormone.

## Data Availability

The data used to support the findings of this study are included within the article.
